# Pre- and Postnatal Exposures to Residential Pesticides and Survival of Childhood Acute Lymphoblastic Leukemia

**DOI:** 10.3390/cancers17060978

**Published:** 2025-03-14

**Authors:** Seema Desai, Libby M. Morimoto, Alice Y. Kang, Mark D. Miller, Joseph L. Wiemels, Lena E. Winestone, Catherine Metayer

**Affiliations:** 1 Division of Epidemiology, School of Public Health, University of California, Berkeley, CA 94720, USA; seema.desai@berkeley.edu (S.D.); libbym@berkeley.edu (L.M.M.);; 2 Division of Occupational, Environmental, and Climate Medicine, University of California, San Francisco, CA 94143, USA; ucsfpehsumiller@gmail.com; 3 Center for Genetic Epidemiology, Department of Population and Public Health Sciences, Keck School of Medicine, University of Southern California, Los Angeles, CA 90033, USA; wiemels@usc.edu; 4 USC Norris Comprehensive Cancer Center, University of Southern California, Los Angeles, CA 90033, USA; 5 Division of Allergy, Immunology, and BMT, Department of Pediatrics, University of California San Francisco Benioff Children’s Hospitals, San Francisco, CA 94158, USA

**Keywords:** childhood acute lymphoblastic leukemia, survival, residential pesticides, rodenticides, developmental periods, pregnancy

## Abstract

**Background:** Exposure to pesticides has been associated with an increased risk of developing childhood leukemia. However, the impact of pesticides on childhood leukemia survival has not been examined. We investigated the associations between residential pesticide use during key developmental periods and 5-year survival in children treated for acute lymphoblastic leukemia (ALL). **Methods:** Residential use of insecticides, herbicides, rodenticides, and flea control products from preconception up to 12 months prior to diagnosis and sociodemographic characteristics were collected via parental interview among 837 children diagnosed with ALL between 1995 and 2008 in California, USA. Data on clinical features were abstracted from medical records. Vital status was obtained through linkage to the National Death Index (NDI) up to 2020. Cox proportional hazards regression models were used to estimate hazard ratios (HRs), adjusting for sociodemographic factors and clinical risk group. **Results:** A total of 108 children with ALL (~13%) died within 5 years of diagnosis. Exposure to any pesticides pre- and/or postnatally was slightly higher among deceased compared to alive children (95.4% vs. 91.5%; *p* = 0.23), while use of rodenticides was significantly higher in children who died (25.0%) vs. those who survived (15.5%; *p* = 0.02). In fully adjusted models, exposure to rodenticides was associated with an increased risk of mortality (HR 1.70; 95% confidence interval (CI) 1.08–2.64; *p* = 0.02), especially when the child was exposed during pregnancy (HR 1.90; 95% CI 1.15–3.16; *p* = 0.01) and possibly 12 months before diagnosis (HR 1.60; 95% CI 0.98–2.61; *p* = 0.06). Increased hazards of death were also observed with other types of pesticides during pregnancy, but those associations were not statistically significant. **Conclusions:** This study is the first to report reduced survival among children with ALL previously exposed to rodenticides, particularly during pregnancy, underscoring the need to further evaluate mechanisms by which environmental exposures during key developmental stages may later impact cancer outcomes.

## Simple Summary

Pesticides have been linked to an increased risk of developing childhood leukemia, yet their impact on survival remains unclear. This study examines whether reported use of pesticides at home before and after birth influences five-year survival in children with acute lymphoblastic leukemia. Our data showed that exposure to pesticides during pregnancy, particularly rodenticides, was linked to a higher risk of death after accounting for other prognostic factors. These findings highlight the need to examine preventable environmental factors that may affect childhood leukemia outcomes, with the goal of improving survival.

## 1. Introduction

In industrialized countries, leukemia stands as the most prevalent malignancy in children, representing 25% of all pediatric cancers. Among its various subtypes, acute lymphoblastic leukemia (ALL) is the dominant contributor, encompassing 78% of all childhood leukemia cases [1]. Despite its relative prevalence, leukemia in children remains a rare disease, with an incidence of 39 cases per million, peaking between the ages of 2 and 5, and exhibiting heightened rates among the Latinx population in the United States [1,2,3]. Childhood leukemia originates from genetic disruptions, often beginning in utero with oncogenic fusion proteins, followed by additional hits postnatally [2].

Pesticides—used to control unwanted plants, insects, and animals—expose children through ingestion, inhalation, and skin contact from home use, agricultural drift, and contaminated food. Chronic low-level pesticide exposure, particularly from residential and occupational sources during pregnancy, is linked to higher childhood leukemia risk [4,5,6,7,8,9,10].

Environmental chemicals may also contribute to cancer initiation and progression via DNA damage, oxidative stress, and immune reactions [2,11,12]. A metanalysis showed that children from lower socioeconomic backgrounds experience a survival gap compared to those from higher socioeconomic backgrounds [13]. Racial disparities also persist, with higher mortality rates among Latinx and Black patients, attributed to factors like genetics, language barriers, and treatment responses [14,15].

Prognostic factors such as socioeconomic status (SES), race, and ethnicity are interlinked with environmental exposures [16], but little is known about the potential independent impact of chemicals on cancer survival. Few studies have examined the link between environmental exposures during perinatal development and pediatric leukemia survival. A Spanish study found maternal smoking during pregnancy and postnatally increased mortality 4-fold, adverse events 8-fold, and treatment-related mortality 14-fold [17]. Data from the California Childhood Leukemia Study (CCLS) showed that paternal preconception smoking and passive smoke exposure reduced 5-year overall survival [18]. Poor perceived air quality and fine particulate matter levels were also associated with lower survival in childhood ALL, lymphomas, and other cancers [19,20,21,22]. The impact of pesticides has been investigated for cancer survival in adults but not children [23,24].

This study leverages data from the CCLS, a case–control study designed to investigate environmental and genetic risk factors for childhood leukemia [18]. We aim to evaluate whether pre- and postnatal residential pesticide exposures influence survival outcomes in children diagnosed with ALL in California.

## 2. Materials and Methods

The CCLS is a case–control study that includes incident cases of childhood leukemia from hospitals across California and matched population-based controls; cases enrolled from 1995 to 2008 were evaluated in this study. Patients with childhood leukemia were enrolled around the time of diagnosis at 17 hospitals if they were younger than 14 years old at diagnosis, had an English or Spanish-speaking parent, lived in one of the study counties at diagnosis, and had no previous cancer. Interviews with a parent, primarily mothers, used a structured questionnaire to collect information on residential use of 12 types of pesticides (yes/no) during three critical developmental periods, including three months before conception, pregnancy, and postnatally (until the child turned three or was diagnosed, whatever occurred first), as well as within a year of the interview following the leukemia diagnosis. The process of extracting sociodemographic data, leukemia type, and vital status through medical record abstraction, clinician validation, and probabilistic linkage to electronic death certificate data was described in a previous study [18]. Of the 837 consenting ALL cases with completed interviews, 108 were linked to death records, with 5 deaths due to external causes.

The primary exposure of interest was pesticide use. Based on their intended pest targets, the 12 pesticide types were grouped into four broad categories: insecticides for controlling various household and lawn insects (5 types), herbicides for targeting various unwanted plants (2 types), flea control for managing fleas on pets and in living areas (4 types), and rodenticides for controlling rodents (1 type). The total number of pesticide types were also categorized into three exposure levels—low (0–2 types), medium (3–4 types), and high (5–12 types)—based on the tertile distribution.

The outcome evaluated was 5-year survival from all causes except external causes. The nonparametric Kaplan–Meier estimator was used to estimate the survival function and survival curves by pesticide exposure group. A directed acyclic graph (DAG) (Figure S1) identified covariates and their relationships with exposure and outcome. Backward elimination further refined the model, excluding birth weight and household dependents due to minimal impact on risk estimates (<10%). The goodness-of-fit tests using Bayesian Information Criterion (BIC) values for the models with and without these covariates are provided in the Supplemental Materials (Table S1). Cox proportional hazards regression was used to estimate hazard ratios (HRs) and 95% confidence intervals (CIs), adjusting for birth year, parental highest education attained (dichotomized—high school or lower vs. some college or more), annual household income (six categories), race and ethnicity (five categories), and National Cancer Institute (NCI) risk group for ALL (categorical: “standard”, defined as age > 1 year and age < 10 years and WBC < 50,000/µL; “high”, defined as age ≥ 10 years or age > 1 year and age < 10 years and WBC ≥ 50,000/µL; and “infant”, defined as age < 1 year) [25]. Adjusted Cox models were run with and without SES variables to assess their potential impact on survival outcomes.

Stratified analyses by breastfeeding duration and race and ethnicity were conducted to explore potential effect modification. Breastfeeding duration was considered due to its known influence on early immune development [26], while race and ethnicity were analyzed to account for potential sociodemographic disparities in health outcomes [15,27]. Heterogeneity in HRs for pesticide groups across critical developmental periods was tested using log-rank tests. Log-likelihood ratio tests were used to assess the goodness-of-fit and effect modification by breastfeeding and race and ethnicity. Analyses were performed in the R environment, version 4.3.1 (16 June 2023) [28]. All tests were two-sided, and *p*-values < 0.05 were considered statistically significant.

This study was approved by the Institutional Review Boards at the University of California, Berkeley, and the California Department of Public Health.

## 3. Results

### 3.1. Population Characteristics

Among 837 children with ALL, 47% were Latinx, 35% were non-Latinx Whites, and the remainder were either Asian/Pacific Islander, Black, or of unknown origin. A total of 131 (16%) children came from households with an income below $15,000, and 129 (15%) children lived in households with more than six dependents. Additionally, 36% of parents had a high school education or less. As presented in Table 1, children who died were more likely to have high-risk ALL, to be diagnosed before the age of one year, and belong to families with low educational attainment and low annual income. Racial disparities were evident, with non-Latinx Black children exhibiting the highest percentage of deceased, followed by Latinx children. There was a suggestion that children who were not breastfed were more likely to die compared to those who were breastfed. The distributions of sex (assigned at birth), number of dependents in the household, birthweight, and gestational age were similar between children who survived and those who did not. Overall, Latinx households and those with low annual income and low education attainment were less likely to use pesticides (Table S2).

### 3.2. Bivariate Analyses

About 92% of all children with ALL were exposed to at least one pesticide type pre- and/or postnatally. The use of pesticides tended to be correlated across different periods. Rodenticide exposure, for instance, showed strong correlations between preconception and pregnancy (correlation coefficient r = 0.82) and between pregnancy and postnatal periods (r = 0.78), suggesting consistent use over time (Figure S2). Conversely, correlations between different pesticide categories were generally low, indicating distinct patterns of use.

**Figure 1:**
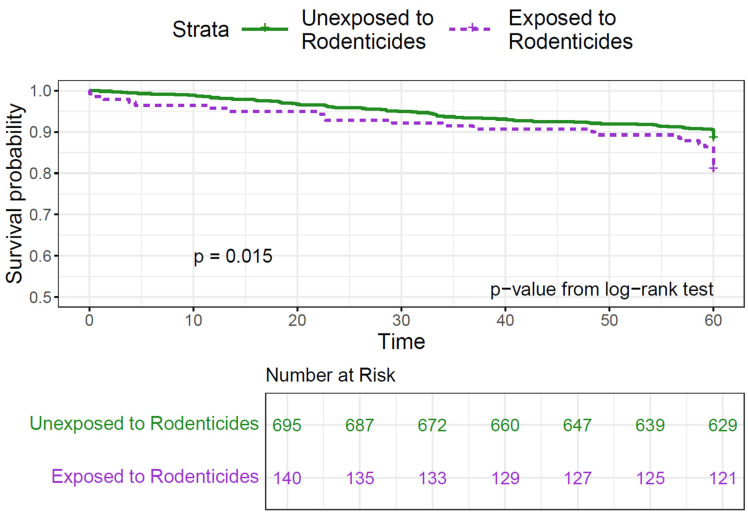
Kaplan–Meier curves for 5-year survival in childhood ALL by rodenticide exposure during any time period.

**Figure 2:**
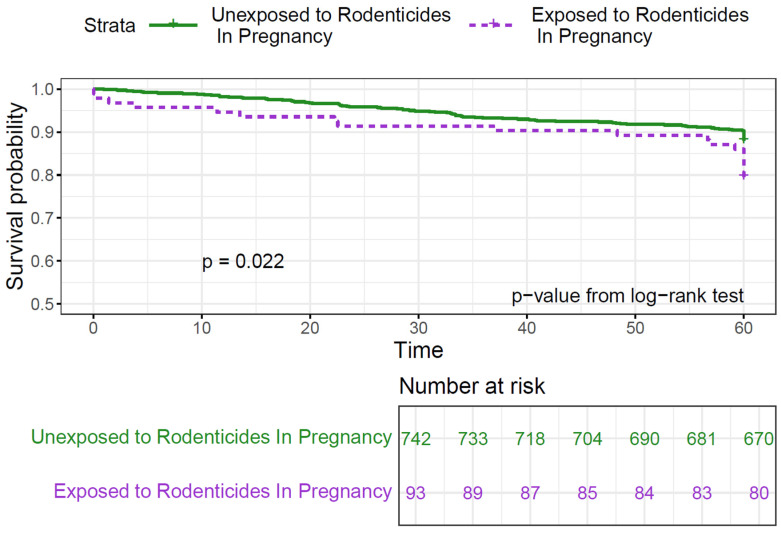
Kaplan–Meier curves for 5-year survival in childhood ALL by rodenticide exposure during pregnancy.

Bivariate analysis showed no significant difference in survival between children ever exposed to pesticides and those never exposed (*p* = 0.23, Table 2). However, survival was lower in children exposed to rodenticides, with 25% exposed among the deceased compared to 15.5% among survivors (*p* = 0.02). Exposure to rodenticides pre- and postnatally was associated with lower survival rates (80–83%) compared to unexposed children (85–90%) or those exposed to other pesticides (Table S3). The Kaplan–Meier curves showed a statistically significant decrease in 5-year survival in the group exposed to rodenticides at any time (*p* = 0.015; Figure 1) and during pregnancy (*p* = 0.022; Figure 2).

### 3.3. Multivariate Analyses

Table 2 shows the HRs for 5-year survival of childhood ALL in relation to pesticide exposure at any time, with adjustment for age at diagnosis, race and ethnicity, and NCI risk group (Model 1) and additional adjustment for SES, including parental education and annual household income (Model 2). A two-fold increased risk of mortality was associated with overall exposure to any pesticides in both models but did not reach statistical significance (*p* = 0.09). Increased risk of mortality was associated with exposure to rodenticides in Model 1 (HR 1.75; 95% CI: 1.13–2.72) and Model 2 (HR 1.69; 95% CI: 1.08–2.64). There was no clear dose–response relationship when examining the number of pesticide types used.

In fully adjusted Cox proportional hazards models examining childhood ALL survival by pesticide exposure during various developmental periods (Table 3), statistically significant increased risks of mortality were seen with exposure to any pesticide during pregnancy (HR 1.6; 95% CI 1.05–2.42), mostly driven by rodenticides (HR 1.91; 95% CI 1.15–3.16) and possibly insecticides and herbicides that conferred 45 to 50% increased risks of mortality, although falling short of statistical significance. Additionally, data suggested that children exposed to rodenticides 12 months prior to the interview had a 60% increased risk of mortality (*p* = 0.06). No significant differences were observed for other pesticide categories across different developmental periods. Adjusting for highly correlated variables challenged model robustness. Sensitivity analysis adjusting across different time windows yielded consistent trends with the primary findings on rodenticide exposure during pregnancy compared to those unexposed (HR 3.12; 95% CI 1.01–9.65) (Table S4). Similarly, rodenticide exposure during pregnancy yielded an HR of 1.74 (95% CI 1.02–2.99) compared to those who were unexposed, after adjusting for other pesticide use during pregnancy (Table S5).

### 3.4. Effect Modification

Stratified analyses by racial and ethnic group showed that rodenticide exposure was associated with poorer survival among non-Latinx White children (HR 3.35; 95% CI 1.42–7.88), while a weaker association was observed in Latinx children (HR 1.66; 95% CI 0.91–3.01) (Table 4). No significant associations were seen in other racial/ethnic groups. Formal testing for effect modification confirmed that survival varied by race and ethnicity (*p* for interaction = 0.02).

Stratified analyses by breastfeeding duration suggested that children exposed to insecticides and who were not breastfed or breastfed for 6 months or less had a higher risk of dying (HR 1.83; 95% CI 0.82–4.08) compared to those unexposed. In contrast, among children breastfed for more than 6 months, insecticide exposure was not associated with increased mortality risk (HR 0.82; 95% CI 0.31–2.12). Formal tests for effect modification, however, did not reach statistical significance (*p* for interaction = 0.11) (Table S6).

## 4. Discussion

### 4.1. Key Findings

Our study, based on comprehensive interview data from Californian families, suggests a significant association between exposure to any residential pesticides during pregnancy and lower survival in children with ALL, after adjusting for clinical and sociodemographic factors. This association was mostly driven by exposure to rodenticides, and to a lesser extent insecticides and herbicides. These findings emphasize the vulnerability of pesticide-exposed patients, highlighting the impact of exposure prior to diagnosis.

To our knowledge, no other childhood cancer study has investigated the relationship between pesticide exposure and survival. Data among adults are also scarce. A 2019 French study examined lymphoma patients with occupational pesticide exposure and found reduced response to immunochemotherapy and lower survival [23]. Another study of Hodgkin’s lymphoma patients residing near agricultural fields in California observed no significant association between environmental pesticide exposure and survival [24]. The lack of convergence among adult studies may be due to differences in routes and levels of exposure to agricultural pesticides.

We examined pesticide exposures during key developmental periods and their effects on leukemia survival rates in children. The pregnancy period was particularly impactful, as mortality was associated with exposure to both any pesticide category and rodenticides. Sensitivity analyses adjusting for collinearity indicated that rodenticide exposure during pregnancy significantly increased the hazard of mortality, highlighting pregnancy as a critical period. Other pesticides showed no significant associations with mortality across different time windows.

In general, the results from Models 1 and 2 (Table 2) were similar, showing little impact from social factors. To account for the additional confounding effects of healthcare access and financial burden on treatment outcomes, we ran sensitivity analyses adjusting for hospital site and number of household dependents, which did not substantially alter the observed association between rodenticide exposure and poor survival.

Latinx households and those with lower income and education levels reported using fewer pesticides than other groups (Table S2), which may reflect differences in household practices, access to pesticide products, or awareness of risks associated with pests. Stratified analyses indicated differences in the association between pesticide exposure and survival, particularly for rodenticide exposure among non-Latinx White children, possibly due to the disproportionately large number of exposed individuals in this group (Table S1). Smaller sample sizes for racial and ethnic groups limit the robustness of these findings, emphasizing the need to further examine socioeconomic disparities and conduct larger-scale studies [13,16,27,29]. In addition, interview data are somewhat limited in characterizing the levels of exposure to pesticides with precision, therefore limiting the interpretation of our results showing no overall dose–response relationship with survival.

Building on evidence linking short breastfeeding duration to childhood leukemia risk [26,30,31,32,33], our data suggest higher pesticide-related survival risks in children breastfed for less than six months. Though interaction tests were not significant, this warrants further study on immune modulators’ roles in cancer relapse and survival, particularly in mitigating the adverse survival effects associated with rodenticide exposure.

### 4.2. Biological Effects of Pesticides

Population studies have consistently linked residential pesticide exposure to an elevated risk of developing childhood leukemia, emphasizing both in utero and postnatal exposures [6,34,35,36]. Like other leukemogenic agents, such as etoposide, benzene metabolites, and lack of bioflavonoids, certain pesticides exert toxicity through oxidative stress and mitochondrial dysfunction. These processes can induce DNA breaks, potentially leading to chromosomal rearrangements (duplications, deletions, and translocations) if not properly repaired [37]. The initial impact often occurs in utero, giving rise to oncogenic fusion proteins. Subsequent insults, determining disease latency, occur post-birth and may involve genetic, epigenetic, or immune factors (e.g., delayed infection-mediated immune deregulation) [2]. Studies suggest that pesticides like organophosphates, carbamates, and pyrethroids—commonly present in insecticides and herbicides—can impair leukocyte function by inducing apoptosis, arresting the cell cycle, and disrupting immune cell functions [38]. Distinct patterns in chromosomal aberrations, cytologic features, and peripheral blood and bone marrow indices (similar to those found in patients with secondary leukemia typically induced by radiation or chemotherapy) have been documented in adult patients with acute myeloblastic leukemia who have been exposed to pesticides (*n* = 21) vs. those not exposed (*n* = 40) [39]. The authors suggested that pesticide exposure may worsen leukemia prognosis and survival by triggering harder-to-treat cytogenetic and clinical subtypes. Overall, epidemiological and biological data support the role of certain pesticides in both the development and prognosis of leukemias.

### 4.3. Rodenticides: Prevalence and Potential Health Risks

Household and agricultural rodenticide use is common, resulting in over 8000 calls to poison centers in 2021 [40]. Most commonly, these calls are related to ingestion, either intentional or unintentional. It is uncertain how often or to what degree incidental small exposures not prompting calls occur. Rodenticides are used in bait stations and have a low risk of volatilization [41,42] that minimizes the likelihood of exposures of acute significance, but undocumented low-dose exposures may occur relatively commonly in the process of opening, moving, or disposing of bait stations. We could not identify any biomonitoring studies examining this kind of low-dose exposure. In addition, there have been many reported cases of illegally imported chemicals used as rodenticides, such as tetramethylenedisulfotetramine and aldicarb, with entirely different mechanisms of action, causing acute illness in the U.S. [43,44]. The degree to which these factors support the biologic plausibility of our findings is uncertain.

Rodenticides include non-anticoagulants like bromethalin, a neurotoxic compound that disrupts oxidative phosphorylation, leading to cytotoxic edema, though human exposure reports suggest it is found in sub-lethal concentrations with no clear dose–response threshold [45,46]. They also contain anticoagulants like brodifacoum, a potent second-generation “super warfarin” that inhibits vitamin K recycling and disrupts blood clotting [47]. It was found in d-Con, the primary rodent-control product used by participants in our study, at a concentration of 0.005% until 2015, when the EPA banned its residential use. However, brodifacoum remains widely used in professional and agricultural settings [48]. Brodifacoum inhibits vitamin K epoxide reductase (VKOR), disrupting the vitamin K cycle, reducing clotting factor synthesis, and prolonging coagulation times [49,50,51]. Known for its high affinity and prolonged elimination half-life, brodifacoum causes acute poisonings with symptoms resembling fatal leukemia [52]. Our study emphasizes the need to investigate rodenticide exposures’ mechanism of effects on leukemia survival, focusing on hematologic and non-hematologic mechanisms tied to vitamin K inhibition [53].

### 4.4. Limitations and Strengths

Our study leveraged existing data on pesticide exposure during key developmental periods from a case-control study, though reliance on self-reported questionnaires may introduce recall bias influenced by parents’ perceptions or societal pressures. However, 73% of ALL cases were diagnosed under the age of 6, likely improving recall accuracy, supported by consistent data across periods, including the reliable 12-month period prior to the interview. High correlation between exposure periods limits our ability to draw definitive conclusions on the relative contributions of prenatal vs. postnatal exposure. While deceased children were not excluded, parents of 50 children who passed away shortly after enrollment did not complete the interview. The survival rate (87%) aligned with national averages (1995–2015) [30,54], and demographic data from the birth registry for these deceased children who did not complete the interview were comparable to the other cases included, supporting representativeness. However, potential differences in neighborhood income between interviewed and non-interviewed families raise concerns about selection bias. Despite adjustments for key sociodemographic factors in our analysis, residual confounding SES factors cannot be ruled out. The causal diagram indicated no need for additional adjustments beyond income, education, and race and ethnicity, and the additional adjustment for hospital sites did not change the results. However, our data did not capture detailed information on access to specialized healthcare, type of medical insurance, or treatment-related factors such as financial resources, drug availability, and access to novel therapies, all of which may influence survival outcomes. Variability in these factors, including timely administration of conventional treatments and management of therapy-related complications, could contribute to disparities in survival [55]. Finally, the limited number of exposed cases, coupled with high correlation of exposure across time periods, constrain the statistical power of our findings and make it challenging to disentangle the independent effect of exposure during a specific time period driving the association.

This study demonstrates key strengths in accounting for potential confounders, largely due to the rich sociodemographic and pesticide exposure data from the CCLS interviews, which provided insights into records of linkage studies, thus enhancing the rigor of our analyses.

## 5. Conclusions

Our study, featuring detailed data collection and attention to confounders, suggests associations between pesticide exposure—especially for use of rodenticides during pregnancy—and reduced childhood ALL survival. Future studies should aim for more direct exposure assessment methods, larger sample sizes, and a more comprehensive evaluation of leukemia prognosis, including molecular subtypes of ALL and treatment response. By situating our work within the broader context of the impact of environmental exposures on the pediatric cancer continuum from etiology to short- and long-term outcomes, we contribute to the growing body of knowledge on the impact of chemical exposures on childhood leukemia prognosis. This study stands as an initial step towards understanding the effects of pesticide exposure during key developmental stages on the survival outcomes of children with leukemia, urging further research to enhance survival outcomes by addressing preventable environmental factors.

## Figures and Tables

**Table 1:** Characteristics of 837 children with acute lymphoblastic leukemia both overall and stratified by 5-year survival status at the end of 2020—the California Childhood Leukemia Study.

Characteristics	Overall *n* = 837	Alive *n* = 729	Deceased *n* = 108
	*n* (%)	*n* (%)	*n* (%)
Sex (assigned at birth)			
Female	366 (43.7)	324 (44.4)	42 (38.9)
Male	471 (56.3)	405 (55.6)	66 (61.1)
Race and Ethnicity			
Latinx	396 (47.3)	340 (46.6)	56 (51.9)
Non-Latinx White	295 (35.2)	272 (37.3)	23 (21.3)
Non-Latinx Asian/Pacific Islander	73 (8.7)	61 (8.4)	12 (11.1)
Non-Latinx Black	24 (2.9)	16 (2.2)	8 (7.4)
Other/Unknown	49 (5.9)		
Birth Years			
1982–1989	64 (7.6)	48 (6.6)	16 (14.8)
1990–1999	509 (60.8)	442 (60.6)	67 (62.1)
2000–2014	264 (31.5)	239 (32.8)	25 (23.1)
Household Annual Income (USD)			
<15,000	131 (15.7)	112 (15.4)	19 (17.6)
15,000–29,999	149 (17.8)	123 (16.9)	26 (24.1)
30,000–44,999	130 (15.5)	112 (15.4)	18 (16.7)
45,000–59,999	122 (14.6)	102 (14.0)	20 (18.5)
60,000–74,999	63 (7.5)	57 (7.8)	6 (5.5)
75,000+	242 (28.9)	223 (30.6)	19 (17.6)
Number of Dependents in the Household			
1–3	184 (22.0)	155 (21.2)	29 (26.9)
4–5	524 (62.6)	459 (63.0)	65 (60.1)
6+	129 (15.4)	115 (15.8)	14 (13.0)
Highest Parental Education Attained			
High School or Lower	303 (36.2)	260 (35.7)	43 (39.8)
Some College or More	533 (63.7)	468 (64.2)	65 (60.2)
Unknown	1 (0.1)		
Age at Diagnosis (years)			
<1	27 (3.2)	13 (1.8)	14 (13.0)
1–2	195 (23.3)	175 (24.0)	20 (18.5)
3–6	391 (46.7)	360 (49.4)	31 (28.7)
7–9	103 (12.3)	86 (11.8)	17 (15.7)
10–14	121 (14.5)	95 (13.0)	26 (24.1)
NCI Risk Group			
Standard	561 (67.0)	509 (69.8)	52 (48.1)
High	226 (27.0)	186 (25.5)	40 (37.0)
Infant	26 (3.1)	12 (1.7)	14 (13.0)
Unknown	24 (2.9)		
Birthweight (grams)			
<2500	39 (4.7)	34 (4.7)	5 (4.6)
2500–4000	663 (79.2)	580 (79.6)	83 (76.9)
>4000	135 (16.1)	115 (15.8)	20 (18.5)
Gestational Age (weeks)			
<36	45 (5.4)	36 (4.9)	9 (8.3)
36–41	608 (72.6)	535 (73.4)	73 (67.6)
41+	175 (20.9)	149 (20.4)	26 (24.1)
Unknown	9 (1.1)		
Breastfeeding			
No	138 (16.5)	114 (15.6)	24 (22.2)
Yes	663 (79.2)	585 (80.2)	78 (72.2)
Unknown	36 (4.3)		
Breastfeeding Duration (months)			
6 or Less	520 (62.1)	450 (61.7)	70 (64.8)
More than 6	281 (33.6)	249 (34.2)	32 (29.6)
Unknown	36 (4.3)		

Percentages may not amount to 100% due to rounding. Abbreviations: USD—United States Dollar; NCI—National Cancer Institute.

**Table 2:** Residential pesticides and 5-year survival in childhood acute lymphoblastic leukemia: Cox proportional hazards models without and with adjustments for socioeconomic status.

Exposure	Alive *n* = 729	Deceased *n* = 108		Model 1—Without SES Adjustment *		Model 2—With SES Adjustment **	
	*n* (%)	*n* (%)	*p*-Value	HR (95% CI)	*p*-Value	HR (95% CI)	*p*-Value
Any Pesticides							
No	62 (8.5)	5 (4.6)		Ref.		Ref.	
Yes	667 (91.5)	103 (95.4)	0.23	2.06 (0.83–5.11)	0.1	2.22 (0.89–5.54)	0.09
*Number of Types Used*							
0–2 (Low)	327 (44.9)	40 (37.0)		Ref.		Ref.	
3–4 (Medium)	226 (31.0)	42 (38.9)		1.67 (1.07–2.59)	0.02	1.77 (1.14–2.77)	0.01
5–12 (High)	176 (24.1)	26 (24.1)	0.17	1.47 (0.88–2.44)	0.14	1.56 (0.93–2.62)	0.09
Insecticides							
No	110 (15.0)	15 (13.9)		Ref.		Ref.	
Yes	619 (84.9)	93 (86.1)	0.86	1.10 (0.63–1.91)	0.7	1.15 (0.66–2.00)	0.6
Herbicides							
No	362 (49.7)	54 (50.0)		Ref.		Ref.	
Yes	367 (50.3)	54 (50.0)	1	1.16 (0.78–1.72)	0.5	1.31 (0.87–1.98)	0.2
Flea Control							
No	419 (57.5)	64 (59.3)		Ref.		Ref.	
Yes	310 (42.5)	44 (40.7)	0.8	1.16 (0.72–1.57)	0.8	1.04 (0.70–1.54)	0.8
Rodenticides							
No	614 (84.2)	81 (75.0)		Ref.		Ref.	
Yes	113 (15.5)	27 (25.0)	0.02	1.75 (1.13–2.72)	0.01	1.69 (1.08–2.64)	0.02
Unknown (*n* = 2)							

Percentages may not amount to 100% due to rounding. Abbreviations: HR—hazards ratio; CI—confidence interval; Ref—reference; SES—socioeconomic status. * Adjusted for age at diagnosis, race and ethnicity, and NCI risk group status. ** Adjusted for age at diagnosis, race and ethnicity, NCI risk group status, highest parental education attained, and household income.

**Table 3:** Residential pesticides and 5-year survival in childhood acute lymphoblastic leukemia: Cox proportional hazards models * by periods of exposure.

Exposures	Preconception		Pregnancy		Postnatally		12 Months Before Interview	
	HR (95% CI)	*p*-Value	HR (95% CI)	*p*-Value	HR (95% CI)	*p*-Value	HR (95% CI)	*p*-Value
Any Pesticides								
No	Ref.		Ref.		Ref.		Ref.	
Yes	1.02 (0.68–1.52)	>0.90	1.60 (1.05–2.42)	0.03	0.84 (0.53–1.35)	0.5	1.35 (0.87–2.09)	0.2
Insecticides								
No	Ref.		Ref.		Ref.		Ref.	
Yes	1.00 (0.67–1.48)	>0.90	1.45 (0.96–2.17)	0.08	0.81 (0.53–1.25)	0.3	1.13 (0.75–1.71)	0.6
Herbicides								
No	Ref.		Ref.		Ref.		Ref.	
Yes	1.22 (0.79–1.88)	0.4	1.50 (0.98–2.29)	0.06	1.10 (0.73–1.65)	0.7	1.26 (0.84–1.90)	0.3
Flea Control								
No	Ref.		Ref.		Ref.		Ref.	
Yes	0.90 (0.57–1.43)	0.7	1.05 (0.68–1.64)	0.8	0.83 (0.55–1.26)	0.4	1.11 (0.74–1.68)	0.6
Rodenticides								
No	Ref.		Ref.		Ref.		Ref.	
Yes	1.49 (0.85–2.63)	0.2	1.91 (1.15–3.16)	0.01	1.46 (0.91–2.33)	0.1	1.60 (0.98–2.61)	0.06

Abbreviations: HR—hazards ratio; CI—confidence interval; Ref—reference. * Adjusted for age at diagnosis, race and ethnicity, highest parental education attained, household income, and NCI risk group status.

**Table 4:** Residential pesticides and 5-year survival in childhood acute lymphoblastic leukemia: Cox proportional hazards models * by race and ethnicity.

Exposure	Non-Latinx White		Latinx		Non-Latinx Black + Asian + Others		Interaction *p*-Value
	HR (95%CI) *n* Total/*n* Deaths	*p*-Value	HR (95% CI) *n* Total/*n* Deaths	*p*-Value	HR (95% CI) *n* Total/*n* Deaths	*p*-Value	
Insecticides	1.02 (0.24–4.41)	>0.90	1.41 (0.69–2.9)	0.3	0.70 (0.22–2.26)	0.6	0.8
	265/21		317/47		130/25		
Herbicides	1.00 (0.41–2.45)	0.9	0.54 (0.88–2.68)	0.1	1.40 (0.59–3.28)	0.4	0.8
	197/15		141/23		83/16		
Flea Control	0.83 (0.36–1.90)	0.7	1.55 (0.91–2.64)	0.1	0.55 (0.22–1.39)	0.2	0.09
	164/12		145/26		45/6		
Rodenticides	3.35 (1.42–7.88)	0.005	1.66 (0.91–3.01)	0.1	0.45 (0.10–1.93)	0.3	0.02
	46/9		73/16		21/2		

Abbreviations: HR—hazards ratio; CI—confidence interval. * Adjusted for age at diagnosis, race and ethnicity, highest parental education attained, household income, and NCI risk group status.

## Data Availability

The epidemiological and clinical data generated in this study are not publicly available due to the terms of the informed consent signed when subjects were enrolled to the CCLS study but may be available upon reasonable request from the corresponding author. The death data analyzed in this study were obtained from the California Department of Public Health (CDPH) Center for Health Statistics and Informatics (CHSI) and are not publicly available due to terms of CDPH-CHSI.

## Supplementary Materials

The following supporting information can be downloaded at: https://www.mdpi.com/article/10.3390/cancers17060978/s1, Table S1: Estimates and goodness-of-fit statistics for model comparison: Cox proportional hazards model for any pesticide exposure among children with acute lymphoblastic leukemia by 5-year survival status at the end of 2020—the California Childhood Leukemia Study; Table S2: Sociodemographic characteristics and pesticide exposure among 837 children with acute lymphoblastic leukemia—the California Childhood Leukemia Study; Table S3: Periods of residential pesticide use by 5-year survival status at the end of 2020 in children with acute lymphoblastic leukemia—the California Childhood Leukemia Study; Table S4. Multivariate analysis of rodenticide exposures adjusted for all time windows among children with acute lymphoblastic leukemia using Cox proportional hazards model by 5-year survival status at the end of 2020—the California Childhood Leukemia Study; Table S5. Multivariate analysis of pesticide exposures in pregnancy period adjusted for other pesticide groups among children acute lymphoblastic leukemia using the Cox proportional hazards model * by 5-year survival status at the end of 2020—the California Childhood Leukemia Study; Table S6. Residential pesticides and 5-year survival at the end of 2020 in childhood acute lymphoblastic leukemia: Cox proportional hazards models * by duration of breastfeeding; Figure S1: Directed acyclic graph (DAG); Figure S2: Correlation matrix between pesticide categories and windows of exposure.

## Abbreviations

The following abbreviations are used in this manuscript:

ALL	Acute Lymphoblastic Leukemia
BIC	Bayesian Information Criterion
CCLS	California Childhood Leukemia Study
CI	Confidence Interval
DAG	Directed Acyclic Graph
HR	Hazards Ratio
NCI	National Cancer Institute
Ref	Reference
SES	Socioeconomic Status
