# Supplementary material for: Pre- and Postnatal Exposures to Residential Pesticides and Survival of Childhood Acute Lymphoblastic Leukemia
Source: Cancers (Basel). 2025 Mar 14;17(6):978. doi: 10.3390/cancers17060978 (PMC11941410; doi:10.3390/cancers17060978)
Supplement: Supplementary file 1 [file cancers-17-00978-s001.zip › CL Survival Pesticides_SM Figure S1.pdf]

Supplementary Materials

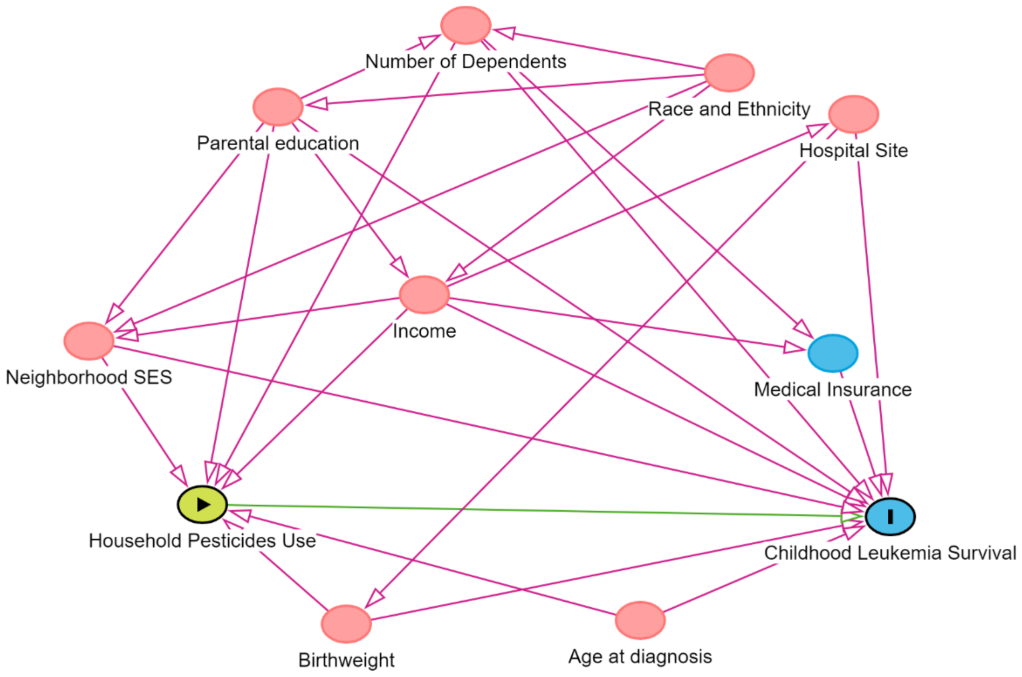

Figure S1: Directed Acyclic Graph (DAG)

Directed Acyclic Graph (DAG) illustrating causal and biasing paths, including ancestral structure, for the relationship between a child's exposure to household pesticide use (Exposure) and acute lymphoblastic leukemia survival (Outcome) in the California Childhood Leukemia Study. The minimal sufficient adjustment set comprises age at diagnosis, birthweight, parental income, neighborhood SES, and number of dependents in a household. Income, race and ethnicity serve as proxy indicators for neighborhood SES in the Cox model for adjustment. Birthweight and the number of dependents in a household were excluded from the final model using the backward elimination method. This DAG was constructed using DAGitty.net software.
