# Supplementary material for: Pre- and Postnatal Exposures to Residential Pesticides and Survival of Childhood Acute Lymphoblastic Leukemia
Source: Cancers (Basel). 2025 Mar 14;17(6):978. doi: 10.3390/cancers17060978 (PMC11941410; doi:10.3390/cancers17060978)
Supplement: Supplementary file 1 [file cancers-17-00978-s001.zip › CL Survival Pesticides_SM Figure S2.pdf]

# Supplementary Materials

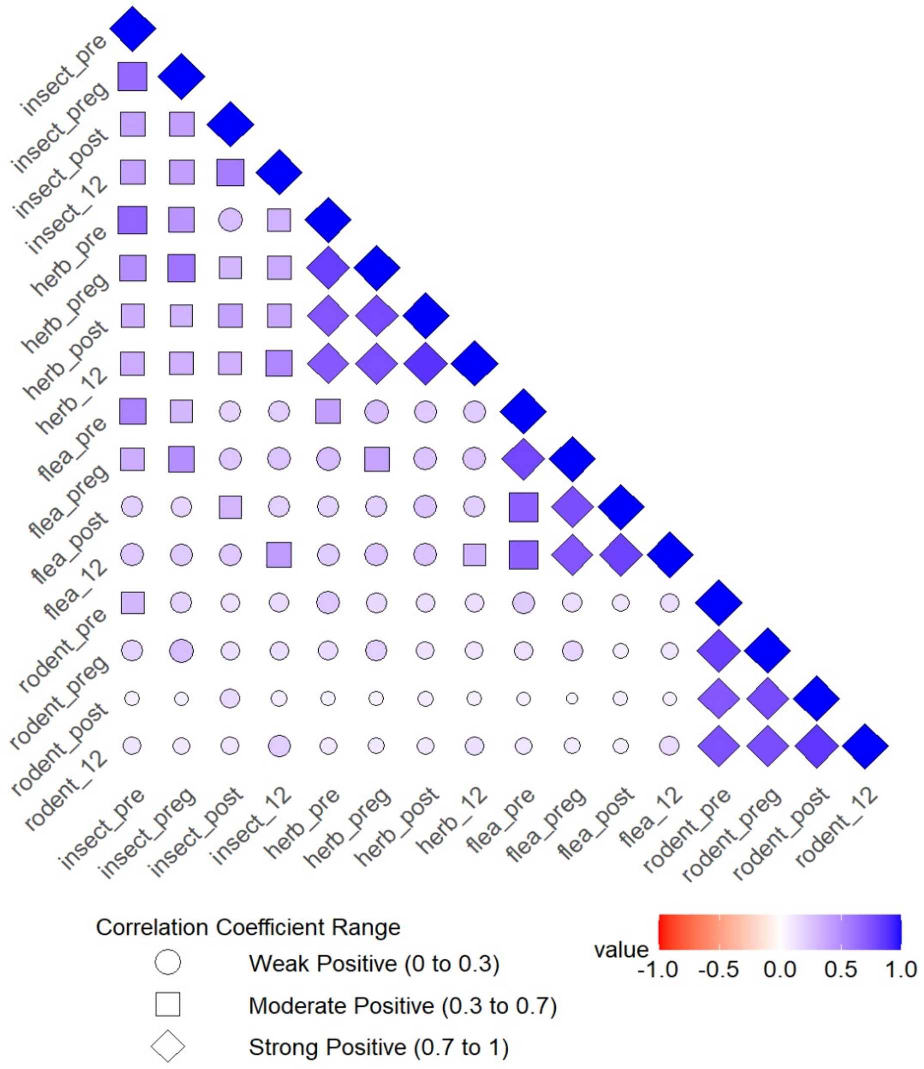


Abbreviations: insect_pre=insecticide exposure before conception; insect_preg=insecticides during pregnancy; insect_postl=insecticides postnatally, insect_12=insecticides in 12-month before interview, herb_pre=herbicides before conception; herb_preg=herbicides during pregnancy; herb_post=herbicides postnatally, herb_12=herbicides in 12 months before interview, flea_pre=flea control before conception; flea_preg=flea control during pregnancy; flea_post=flea control postnatally, flea_12=flea control in 12 months before interview, rodent_pre=rodenticides before conception; rodent_preg=rodenticides during pregnancy; rodent_postl=rodenticides postnatally, rodent_12=rodenticides exposure in 12 months before interview

**Figure S2: Correlation Matrix between Pesticide Categories and Windows of Exposure**

The figure displays Pearson correlation coefficients between different pesticide's exposure levels before conception, during pregnancy, postnatal stages and 12-month period before the interview. The color and size of the circles represent the strength and direction of the correlation, as indicated in the legend at the bottom. A darker blue signifies a stronger positive correlation, whereas a lighter shade indicates a weaker correlation and red indicates no correlation.
