# Supplementary material for: Pre- and Postnatal Exposures to Residential Pesticides and Survival of Childhood Acute Lymphoblastic Leukemia
Source: Cancers (Basel). 2025 Mar 14;17(6):978. doi: 10.3390/cancers17060978 (PMC11941410; doi:10.3390/cancers17060978)
Supplement: Supplementary file 1 [file cancers-17-00978-s001.zip › CL Survival Pesticides_SM Table S1.pdf]

Supplementary Materials

Table S1. Estimates and Goodness-of-Fit Statistics for Model Comparison: Cox Proportional Hazards Model for Any Pesticides Exposure Among Children with Acute Lymphoblastic Leukemia by 5-year Survival Status at the End of 2020: the California Childhood Leukemia Study

Model	HR (95%CI)	P-value	BIC
Final Model* + Birthweight + Number of Dependents in a Household	2.22 (0.89–5.52)	0.09	1480.443
Final Model* + Birthweight	2.22 (0.89–5.55)	0.09	1473.385
Final Model*	2.22 (0.89–5.54)	0.09	1469.429**

Abbreviations: HR: hazards ratio; CI: confidence interval; BIC: Bayesian Information Criterion
*Adjusted for age at diagnosis, race and ethnicity, highest parental education attained, income, and NCI risk group status.
** Lower BIC values indicate a better fit
