# Supplementary material for: Pre- and Postnatal Exposures to Residential Pesticides and Survival of Childhood Acute Lymphoblastic Leukemia
Source: Cancers (Basel). 2025 Mar 14;17(6):978. doi: 10.3390/cancers17060978 (PMC11941410; doi:10.3390/cancers17060978)
Supplement: Supplementary file 1 [file cancers-17-00978-s001.zip › CL Survival Pesticides_SM Table S2.pdf]

Supplementary Materials

Table S2. Sociodemographic Characteristics and Pesticide Exposure Among 837 Children with Acute Lymphoblastic Leukemia: the California Childhood Leukemia Study

Characteristics	Overall n=837	Unexposed n=67	Exposed n=770
	n (%)	n (%)	n (%)
Race and Ethnicity			
Latinx	396 (47.3)	54 (80.6)	342 (44.4)
Non-Latinx White	295 (35.2)	7 (10.5)	288 (37.4)
Non-Latinx Asian/Pacific Islander	73 (8.7)	4 (6.0)	69 (9.0)
Non-Latinx Black	24 (2.9)	1 (1.5)	23 (3.0)
Other/ Unknown	49 (5.9)	1 (1.5)	48 (6.2)
Household Annual Income (USD)			
< 15000	131 (15.7)	22 (32.8)	109 (14.2)
15,000–29,999	149 (17.8)	15 (22.4)	134 (17.4)
30,000–44,999	130 (15.5)	11 (16.4)	119 (15.5)
45,000–59,999	122 (14.6)	9 (13.4)	113 (14.7)
60,000–74,999	63 (7.5)	2 (3.0)	61 (7.9)
75,000+	242 (28.9)	8 (11.9)	234 (30.4)
Highest Parental Education Attained			
High School or Lower	303 (36.2)	47 (70.2)	256 (33.3)
Some College or More	533 (63.7)	20 (29.9)	513 (66.6)
Unknown	1 (0.1)		

Percentages may not sum to 100% due to rounding.
Abbreviation: USD: United States Dollar
