# Supplementary material for: Pre- and Postnatal Exposures to Residential Pesticides and Survival of Childhood Acute Lymphoblastic Leukemia
Source: Cancers (Basel). 2025 Mar 14;17(6):978. doi: 10.3390/cancers17060978 (PMC11941410; doi:10.3390/cancers17060978)
Supplement: Supplementary file 1 [file cancers-17-00978-s001.zip › CL Survival Pesticides_SM Table S3.pdf]

Supplementary Materials

Table S3. Periods of Residential Pesticide Use by 5-Year Survival Status at the End of 2020 in Children with Acute Lymphoblastic Leukemia: the California Childhood Leukemia Study

Exposure	Alive n=729	Deceased n=108	
	n (%)	n (%)	P-value
**Any Pesticides**			
Preconception			
No	332 (45.5)	54 (50.0)	0.44
Yes	397 (54.5)	54 (50.0)	
Pregnancy			
No	290 (39.8)	36 (33.3)	0.24
Yes	439 (60.2)	72 (66.7)	
Postnatal			
No	126 (17.3)	24 (22.2)	0.27
Yes	603 (82.7)	84 (77.8)	
12Months Before Interview			
No	209 (28.7)	30 (27.8)	0.94
Yes	520 (71.3)	78 (72.2)	
**Insecticides**			
Preconception			
No	353 (48.4)	57 (52.8)	0.41
Yes	376 (51.6)	51 (47.2)	
Pregnancy			
No	307 (42.1)	40 (37.0)	0.35
Yes	422 (57.9)	68 (63.0)	
Postnatal			
No	167 (22.9)	31 (28.7)	0.18
Yes	562 (77.1)	77 (71.3)	
12 months Before Interview			
No	238 (32.7)	37 (34.3)	0.74
Yes	491 (67.3)	71 (65.7)	

**Herbicides**			
Preconception			
No	466 (63.9)	71 (65.7)	0.75
Yes	263 (36.1)	37 (34.3)	
Pregnancy			
No	448 (61.4)	64 (59.3)	0.67
Yes	281 (38.6)	44 (40.7)	
Postnatal			
No	376 (51.6)	60 (55.6)	0.47
Yes	353 (48.4)	48 (44.4)	
12 Months Before Interview			
No	405 (55.6)	61 (56.5)	0.92
Yes	324 (44.4)	47 (43.5)	
**Flea Control**			
Preconception			
No	519 (71.2)	83 (76.9)	0.25
Yes	210 (28.8)	25 (23.1)	
Pregnancy			
No	509 (69.8)	78 (72.2)	0.65
Yes	220 (30.2)	30 (27.8)	
Postnatal			
No	435 (59.7)	72 (66.7)	0.17
Yes	294 (40.3)	36 (33.3)	
12 Months Before Interview			
No	477 (65.4)	72 (66.7)	0.83
Yes	252 (34.6)	36 (33.3)	
**Rodenticides**			
Preconception			
No	658 (90.3)	94 (87.0)	0.31
Yes	69 (9.5)	14 (13.0)	
Unknown (n=2)			
Pregnancy			
No	653 (89.6)	89 (82.4)	0.03
Yes	74 (10.1)	19 (17.6)	
Unknown (n=2)			
Postnatal			
No	618 (84.8)	85 (78.7)	0.12
Yes	109 (15.0)	23 (21.3)	
Unknown (n=2)			

12 Months Before Interview			
No	635 (87.1)	88 (81.5)	0.13
Yes	92 (12.6)	20 (18.5)	
Unknown (n=2)			

Percentages may not sum to 100% due to rounding.
