# Supplementary material for: Pre- and Postnatal Exposures to Residential Pesticides and Survival of Childhood Acute Lymphoblastic Leukemia
Source: Cancers (Basel). 2025 Mar 14;17(6):978. doi: 10.3390/cancers17060978 (PMC11941410; doi:10.3390/cancers17060978)
Supplement: Supplementary file 1 [file cancers-17-00978-s001.zip › CL Survival Pesticides_SM Table S4.pdf]

Supplementary Materials

Table S4. Multivariate analysis of Rodenticide Exposures Adjusted for All Time Windows Among Children with Acute Lymphoblastic Leukemia using Cox Proportional Hazards Model* by 5-year Survival Status at the End of 2020: the California Childhood Leukemia Study

Time Windows	HR (95% CI)	P-value
Preconception	0.58 (0.22–1.50)	0.30
Pregnancy	3.12 (1.01–9.65)	0.05
Postnatal	0.68 (0.23–1.98)	0.50
12 Months Before Interview	1.40 (0.50–3.93)	0.50

Abbreviations: HR: hazards ratio; CI: confidence interval
*****Adjusted for rodenticide exposures in other time windows, age at diagnosis, race and ethnicity, highest parental education attained, income, and NCI risk group status
