# Supplementary material for: Pre- and Postnatal Exposures to Residential Pesticides and Survival of Childhood Acute Lymphoblastic Leukemia
Source: Cancers (Basel). 2025 Mar 14;17(6):978. doi: 10.3390/cancers17060978 (PMC11941410; doi:10.3390/cancers17060978)
Supplement: Supplementary file 1 [file cancers-17-00978-s001.zip › CL Survival Pesticides_SM Table S5.pdf]

Supplementary Materials

Table S5. Multivariate analysis of Pesticide Exposures in Pregnancy Period Adjusted for Other Pesticide Groups Among Children Acute Lymphoblastic Leukemia using Cox Proportional Hazards Model* by 5-year Survival Status at the End of 2020: the California Childhood Leukemia Study

Exposure	HR (95% CI)	P-value
Insecticides	1.22 (0.72–2.06)	0.50
Herbicides	0.79 (0.49–1.28)	0.30
Flea Control	1.31 (0.79–2.18)	0.30
Rodenticides	1.74 (1.02–2.99)	0.04

Abbreviations: HR: hazards ratio; CI: confidence interval
*Adjusted for other pesticide group exposures in pregnancy, age at diagnosis, race and ethnicity, highest parental education attained, household income, and NCI risk group status.
