# Supplementary material for: Pre- and Postnatal Exposures to Residential Pesticides and Survival of Childhood Acute Lymphoblastic Leukemia
Source: Cancers (Basel). 2025 Mar 14;17(6):978. doi: 10.3390/cancers17060978 (PMC11941410; doi:10.3390/cancers17060978)
Supplement: Supplementary file 1 [file cancers-17-00978-s001.zip › CL Survival Pesticides_SM Table S6.pdf]

Supplementary Materials

Table S6. Residential Pesticides and 5-Year Survival at the End of 2020 in Childhood Acute Lymphoblastic Leukemia: Cox Proportional Hazards Models* by Duration of Breastfeeding

Exposure	Breastfeeding ≤ 6 Months (n total=520/n deaths=70)		Breastfeeding > 6 Months (n total=281/n deaths=32)		Interaction P-value
	HR (95% CI)	P-value	HR (95% CI)	P-value	
Any Pesticides	4.15 (1.00–17.3)	0.05	2.55 (0.34–19.4)	0.40	0.65
Insecticides	1.83 (0.82–4.08)	0.10	0.82 (0.31–2.12)	0.70	0.11
Herbicides	1.39 (0.83–2.31)	0.20	2.02 (0.9–4.54)	0.10	0.60
Flea Control	1.14 (0.70–1.83)	0.60	0.77(0.34–1.74)	0.50	0.37
Rodenticides	1.84 (1.06–3.19)	0.03	1.40 (0.60–3.29)	0.40	0.78

Abbreviations: HR: hazards ratio; CI: confidence interval
*Adjusted for age at diagnosis, race and ethnicity, highest parental education attained, household income, and NCI risk group status.
